# Physiological Effects of High-Flow Nasal Cannula Therapy and Its Use in Acute Cardiogenic Pulmonary Edema

**DOI:** 10.7759/cureus.13372

**Published:** 2021-02-16

**Authors:** Prakash Adhikari, Sanket Bhattarai, Ashish Gupta, Eiman Ali, Moeez Ali, Mohamed Riad, Jihan A Mostafa

**Affiliations:** 1 Internal Medicine, California Institute of Behavioral Neurosciences & Psychology, Fairfield, USA; 2 Internal Medicine, Piedmont Athens Regional Medical Center, Athens, USA; 3 Research, California Institute of Behavioral Neurosciences & Psychology, Fairfield, USA; 4 Pediatrics, California Institute of Behavioral Neurosciences & Psychology, Fairfield, USA; 5 Psychiatry, California Institute of Behavioral Neurosciences & Psychology, Fairfield, USA

**Keywords:** oxygen therapy, acute cardiogenic pulmonary edema, heart failure, acute hypoxic respiratory failure, high-flow nasal cannula, non-invasive positive-pressure ventilation

## Abstract

High-flow nasal cannula (HFNC) is an open oxygen delivery system, which provides heated and humidified oxygen at a high flow (up to 60 L/min). This effect can improve mucociliary function, airway clearance, and level of comfort to the patient. It can provide controlled and adequate fraction of inspired oxygen (FiO_2_) between 21% and 100%. Generation of end-expiratory pressure helps in carbon dioxide washout, reduction of anatomical dead space, and recruitment of collapsed alveoli, ultimately improving tissue oxygenation.

The use of HFNC in acute hypoxemic respiratory failure, post-extubation period, pre-intubation period, respiratory infection, and obstructive airway disease has been extensively studied, but there are very few studies regarding its use in cardiogenic pulmonary edema. This review provides the current understanding of the physiological effect of HFNC and its application in acute cardiogenic pulmonary edema (ACPE). We conducted a literature search on PubMed using appropriate terms and reviewed relevant articles published within the last 10 years. We found that initial therapy with HFNC in ACPE patients can improve oxygenation and respiratory rate. HFNC can potentially be an alternative to non-invasive positive-pressure ventilation in terms of initial oxygen therapy in patients with ACPE. There is a need for larger prospective studies to evaluate and develop guidelines to consider the use of HFNC in patients with ACPE. We also highlight the fact that if there is no improvement in arterial blood gas parameters after HFNC therapy, initiation of invasive ventilation should not be delayed.

## Introduction and background

Acute cardiogenic pulmonary edema (ACPE) is a major and serious complication of acute heart failure. It has an estimated mortality rate of 10% in hospitalized patients and a one-year mortality rate as high as 30% [1]. It is the second most common parenchymal cause of respiratory failure after pneumonia. Overall, 90% of patients present with dyspnea, while the remaining present with significant respiratory failure in the form of hypoxemia, hypercapnia, and acidosis [2,3]. The initial treatment includes supplemental oxygen, diuretics, vasodilators, non-invasive positive-pressure ventilation (NIPPV) or endotracheal intubation.

Conventional oxygen therapy (COT) with nasal cannula or face mask is the most common method of oxygen delivery. Oxygen supplementation with COT may not always be sufficient and effective. Often there is a need for NIPPV in the form of continuous positive airway pressure (CPAP), bilevel positive airway pressure (BPAP), or even endotracheal intubation [4]. COT cannot provide adequate fraction of inspired oxygen (FiO_2_) if patients’ inspiratory flow rate is higher than the oxygen flow from COT. The additional flow is recruited from atmospheric air (FiO_2_-21) which lowers the delivered FiO_2_. When the oxygen flow is greater than 6 L/min through COT, even bubble humidifier cannot humidify the oxygen sufficiently, which causes dryness of the respiratory tract [5]. The European Society of Cardiology recommends NIPPV for patients with acute and chronic heart failure whose respiratory rate is more than 20 breaths/min as an initial therapy to reduce hypercapnia and acidosis [6]. High-flow nasal cannula (HFNC) is being increasingly used currently for acute respiratory failure due to hypoxemia, post-extubation period, pre-intubation period, respiratory infection, and obstructive airway disease. It can deliver a mixture of air and oxygen at a very high flow rate (maximal flow of 60 L/min). The air is heated and humidified and FiO_2_ can be better regulated [7]. It has been thought that a small positive end-expiratory pressure (PEEP) delivered by the system helps in alveolar recruitment and increased oxygenation of tissues [8]. Some studies have suggested that the use of HFNC reduces nasopharyngeal resistance and pharyngeal dead space [9,10].

Studies have also shown that HFNC can be a potential therapeutic option in patients with acute respiratory failure. The use of HFNC in respiratory failure specifically due to ACPE has not been studied extensively. Do the physiological effects of HFNC provide better outcome in the management of ACPE? Can it be used as an alternative to NIPPV with better outcome in terms of patients’ compliance? We reviewed the mechanism of the physiologic effect of HFNC in respiratory system and searched all available articles published on PubMed within the last 10 years. Research in this field is new and there are no established guidelines for the use of HFNC in ACPE. This review aims to summarize and discuss the current understanding of the physiologic effect of HFNC and its application in ACPE.

## Review

Physiological effects of high-flow nasal cannula

Humidification of Oxygen and Improvement in Mucociliary Clearance

Inhalation of cold and dry air causes dryness of airways, which can impair mucociliary functions such as secretion clearance and airway defense [11]. Dry airways increase airway resistance and work of breathing. HFNC not only provides high flow but also contains an active heated humidifier which warms and humidifies air before delivering it to the airways [12]. A study by Chikata et al. found that 100% humidification can be maintained at high flow (20-50 L/min) at normal body temperature (37˚C) [13]. The humidified air loosens the thick secretions, improves mucociliary functions, and improves airway clearance [14].

Reduction of Anatomical Dead Space and Increased Carbon Dioxide Washout

Anatomical dead space is defined as “the segments of the respiratory tract responsible for conducting air to the alveoli and respiratory bronchioles which does not take part in gas exchange” [12]. Studies have shown high flow produced by HFNC reduces respiratory rate in patient with moderate-to-severe respiratory distress, which in turn decreases minute ventilation (respiratory rate × tidal volume), while partial pressure of carbon dioxide remains steady or even decreases. A retrospective study by Jeong et al. demonstrated similar results in patients with hypercapnia who were treated with HFNC in the emergency department [15]. This proves that continuous flow of fresh air at high flow produces a more efficient ventilation by facilitating carbon dioxide clearance from the anatomical dead space [16,17].

Control in Fraction of Inspired Oxygen

The inspiratory flow of a patient varies with each breath. If inspiratory flow is higher than the supplemental oxygen flow, especially with COT, then the remaining air is recruited from the atmosphere. This dilutes the oxygen content and decreases FiO_2_. HFNC provides high and constant flow and overcomes the issue of gas dilution [18]. FiO_2_ is more constant at higher flows and can be maintained between 0.21 and 1 [19,20].

Generation of Positive Expiratory Pressure and Alveolar Recruitment

NIPPV is a closed system whereas HFNC is an open system. The high flow through HFNC can provide a small positive pressure in the airways. It has been postulated that opening of the mouth during breathing causes escape of gas, thus reducing the pressure in the airway; however, with mouth closed during breathing and a maximum flow of 60 L/min, the pharyngeal pressure remains around 3 cm of water [15,21]. A study by Corley et al. showed a linear relationship between end-expiratory lung volume (EELV) and flow rate. The study showed 0.7% increase in EELV with each liter increase in flow [22]. The PEEP shifts fluid in the alveolar space to perivascular interstitial space and increases the number of alveoli participating in ventilation, which is also known as alveolar recruitment [23,24].

Reduction in Work of Breathing

Inspiration is an active process whereas expiration is a passive process. Respiratory muscles consume energy during inspiration which is also known as work of breathing [12]. One of the main functions of NIPPV is to assist respiratory muscles by reducing work of breathing. Studies have shown that the use of HFNC can reduce work of breathing in infants with respiratory distress and in adults with chronic obstructive pulmonary disease [25,26]. HFNC reduces respiratory rate and improves thoraco-abdominal synchrony during breathing, reducing work of breathing.

Improvement in Patient Comfort

HFNC does not have unwanted effects as observed with nasal or facial mask. Patients can talk and eat while using HFNC. Patients often prefer HFNC to other form of NIPPV (BPAP, CPAP) because they do not have to wear tight-fitting masks which can be uncomfortable. Compared to COT, it is better tolerated because of warmed, humidified gases that do not dry respiratory mucosa [2,27]. Most patients are comfortable using it for a longer period. A summary of the physiological effects of HFNC is presented in Table 1.

**Table 1:** Summary of the physiological effects of HFNC. FiO_2_, inspired oxygen; HFNC, high-flow nasal cannula

Physiological effects of HFNC
Humidification of oxygen and improvement in mucociliary clearance
Reduction of anatomical dead space and increased carbon dioxide washout
Control in fraction of FiO_2_
Generation of positive expiratory pressure and alveolar recruitment
Reduction in work of breathing
Improvement in patient comfort

Adverse effects of high-flow nasal cannula

In the absence of well-established evidence or criteria, clear contraindication for HFNC is lacking. Some studies have suggested avoiding HFNC in patients for whom NIPPV is contraindicated [14]. However, in fact, HFNC can be an alternative when NIPPV is contraindicated, for example, in patients with claustrophobia and intolerance towards tight interface contact. Although HFNC can cause abdominal distention and barotrauma as other NIPPV, the incidence is very rare [12]. There is no guideline for when to escalate respiratory therapy from HFNC to the invasive form. It depends on the judgement of the physician. Between 30% and 40% of patients with acute hypoxic respiratory failure require invasive mechanical ventilation [20]. Delayed intubation is associated with increased mortality in pneumonia [28,29]. Prolonged spontaneous breathing with HFNC may worsen the nature and extent of the initial injury, which is also known as self-inflicted injury. Oxygen supplementation may rapidly normalize oxygen saturation leading to the misjudgment that the patient is improving [30].

Application of high-flow nasal cannula in acute cardiogenic pulmonary edema

The use of HFNC in acute respiratory failure due to various underlying causes has been extensively studied since its introduction. The investigation is more extensive in acute hypoxemic respiratory failure, post-extubation period, pre-intubation period, respiratory infection, and obstructive airway disease [1]. There are very few studies regarding the use of HFNC in respiratory failure due to ACPE. In acute heart failure patients where there is elevated preload and afterload, the use of HFNC can increase cardiac output by reducing both preload and afterload [31,32]. HFNC therapy also reduces intrapulmonary shunting [33]. It provides some degree of PEEP, which increases the intrathoracic pressure. This can cause some degree of inspiratory collapse of inferior vena cava from the patient’s baseline and decrease preload. Roca et al. demonstrated this finding in a sequential interval study on 10 patients with New York Heart Association (NYHA) classification III heart failure where the collapse of inferior vena cava was measured by echocardiogram [34]. The use of CPAP has been the first line oxygen therapy for pulmonary edema because of its ability to improve oxygenation and reduce afterload [35]. A similar effect is exhibited by HFNC with less discomfort, which can be an alternative for patients who are not able to tolerate NIPPV. However, more data is needed to show its effectiveness in this population.

One of the randomized controlled trials by Ko et al. compared the use of COT with HFNC in 69 heart failure patients with pulmonary edema presenting to the emergency department (ED). They obtained respiratory rate, oxygen saturation (SpO_2_), lactate levels and arterial blood gas (ABG) parameters at baseline and 30 and 60 minutes. The result showed improvement in all parameters with HFNC therapy compared with conventional therapy [36]. In terms of ABG parameters, there were significant differences in the PaO_2_ and SpO_2_. They recommended to consider invasive ventilation if there is no significant improvement in ABG parameters within 30 minutes of HFNC [36]. Another randomized controlled trial by Makdee et al. included 128 ED patients with cardiogenic pulmonary edema who were randomized into COT and HFNC. The results of the study showed that 60-min respiratory rate was significantly lower with HFNC than COT [37]. They concluded that HFNC could deliver effective oxygenation and comfort with minimal complications or life-threatening adverse events [37].

Sener et al. conducted a prospective observational study in patients with tachypneic, hypoxemic, hypertensive pulmonary edema. Out of 112 patients, 50 underwent standard oxygen therapy and 62 received high-flow oxygen therapy (HFOT). They studied patient’s zero, first, and second-hour blood gas result and vital signs, requirement of endotracheal intubation, length of hospitalization, and prognosis [4]. The result showed that HFNC can shorten the length of stay both in the ED and intensive care unit. The second-hour lactate levels were significantly lower in the HFOT group suggesting effective tissue and cell oxygenation. HFNC is more effective in patients with hypertensive pulmonary edema than COT in terms of blood gas analysis, heart rate, and respiratory rate in the follow-up period [4]. A cohort study by Chang et al. showed no significant difference in treatment failure within 72 hours among the HFNC and NPPV group [38]. They analyzed 104 patients out of which 58 were in the HFNC group and 46 in the NPPV group. The study concluded that among the patients with heart failure, HFNC was not inferior to NIPPV for preventing extubation failure and reintubation [38]. Studies comparing HFNC with NIPPV/COT are summarized in Table 2.

One of the studies reported that HFNC therapy did not show more benefits compared to COT with respect to endotracheal intubation within 24 hours, intensive care unit admission rate, and 28-day mortality in heart failure patients with acute pulmonary edema [37]. In the study brain natriuretic peptide (BNP) value was statistically higher in the HFNC group than in the COT group. The BNP value increases when left ventricular systolic function decreases [27]. The higher value of BNP is proportional to the severity of heart failure according to the NYHA classification. As the BNP value was significantly higher in the HFNC group than in the COT group, HFNC may have greater degree of severity of heart failure.

**Table 2:** Studies on use of HFNC in ACPE showing study population, intervention/experimental group, control group, and outcome. HFNC, high-flow nasal cannula, COT, conventional oxygen therapy; NIPPV, non-invasive positive-pressure ventilation; ABG, arterial blood gas; SpO_2_, oxygen saturation; PaO_2_, partial pressure of oxygen; n, number of participants; ACPE, acute cardiogenic pulmonary edema

Study	Study population	Intervention/Experimental group	Control group	Outcome
Ko et al. [36] (Randomized controlled trial)	Patient suspected of pulmonary edema due to heart failure	HFNC (n = 36)	COT (n = 33)	Significant difference in respiratory rate, SpO_2_ at 30 and 60 minutes (improved in HFNC group) Significant difference in ABG parameters (PaO_2_ and SpO_2_) at 30 and 60 minutes (improved in HFNC group)
Makdee et al. [37] (Randomized controlled trial)	ED patients with cardiogenic pulmonary edema	HFNC (n = 63)	COT (n = 65)	60-minute respiratory rate significantly lower in HFNC group Lower respiratory rate at 15 and 30 minutes in HFNC group
Sener et al. [4] (Prospective observational study)	Patients with hypertensive pulmonary edema	HFNC (n = 62)	COT (n = 50)	HFNC shortens the length of stay in both emergency and intensive care unit HFNC shows better results in terms of heart rate, respiratory rate, and ABG parameters
Chang et al. [38] (Cohort study)	Post-extubated patients with heart failure with ejection fraction <50%	HFNC (n = 58)	NIPPV (n = 46)	No significant difference in treatment failure between two groups in 72 hours

## Conclusions

Whether the use of HFNC provides an improved outcome in ACPE is a subject of interest. The use of HFNC in acute respiratory failure secondary to acute hypoxemic respiratory failure, post-extubation period, pre-intubation period, respiratory infection, and obstructive airway disease has been studied extensively. However, the data on the use of HFNC in ACPE is scarce. HFNC therapy provides heated and humidified oxygen, which is better tolerated by most patients. Generation of positive expiratory pressure, carbon dioxide washout, reduction in anatomical dead space, alveolar recruitment, and ability to deliver desired FiO_2_ helps in better oxygenation in patients with ACPE. Reduction of preload and afterload in the heart makes it a potential initial therapy in ACPE. It can potentially replace COT and act as an alternative to NIPPV as an initial effective oxygen therapy in patients with ACPE.

There is a need for larger prospective comprehensive studies to evaluate and develop guidelines to determine when invasive ventilation is appropriate in patients with cardiogenic pulmonary edema on HFNC therapy. If there is no improvement in ABG parameters after HFNC therapy, the physician should consider invasive ventilation.
